# Immunological Markers Associated with Skin Manifestations of EGPA

**DOI:** 10.3390/ijms26157472

**Published:** 2025-08-02

**Authors:** Silvia Brunetto, Federica Buta, Sebastiano Gangemi, Luisa Ricciardi

**Affiliations:** Department of Clinical and Experimental Medicine, School and Unit of Allergy and Clinical Immunology, “G. Martino” Hospital, University of Messina, 98124 Messina, Italy; silviabrunetto90@gmail.com (S.B.); federicabuta@gmail.com (F.B.); gangemis@unime.it (S.G.)

**Keywords:** eosinophilic granulomatosis with polyangiitis, skin manifestations, eosinophilia, ANCA, ANA, Mepolizumab, purpura, angioedema, urticarial vasculitis, nodules

## Abstract

Eosinophilic Granulomatosis with Polyangiitis (EGPA) is a rare systemic vasculitis with eosinophilic inflammation and variable clinical presentations. Although skin manifestations are frequent, current classification criteria do not include them, which may underestimate their diagnostic value. This prospective observational study aimed to assess systemic and skin involvement as well as eosinophilia, anti-neutrophil cytoplasmic antibody (ANCA), and Anti-nuclear antibodies (ANA) serum levels in 20 EGPA patients followed for one year at the University Hospital of Messina, Italy, before starting Mepolizumab, 300 mg. Eosinophilia, ANCA status, systemic and skin involvement were also evaluated at 6 and 12 months; a literature review on these data supplements our findings. Skin involvement was present in 55% of patients, including purpura, urticarial vasculitis, angioedema, maculopapular rash, and nodules, mostly in ANCA-negative patients, though purpura was more frequent in ANCA-positive cases but without any statistically significant correlation. ANAs were present in 50% of patients, together with ANCA in two subjects and without in eight. Mepolizumab significantly reduced eosinophil levels, BVASs, and corticosteroid dependence, with notable improvement in skin symptoms. In conclusion, skin manifestations are common in EGPA and may represent useful indicators of disease activity. Their integration with ANCA status, eosinophil counts, and positivity to other autoantibodies could enhance diagnostic and monitoring strategies identifying different clusters of EGPA patients even if the small sample size limits the generalizability of the findings.

## 1. Introduction

Eosinophilic Granulomatosis with Polyangiitis (EGPA), formerly known as Churg–Strauss Syndrome, is a rare systemic necrotizing autoimmune vasculitis of medium-sized vessels [1]. The global incidence of EGPA ranges between 0.5 and 4.2 cases per million individuals per year, with a prevalence estimated at 10 to 14 cases per million [2,3]. Epidemiological studies have not identified significant differences based on sex, geographic distribution, or familial aggregation while pediatric cases seem exceedingly rare [2,4].

EGPA occupies a distinct position within the spectrum of anti-neutrophil cytoplasmic antibody (ANCA)-associated vasculitis (AAV) [5], with eosinophils serving as key effector cells responsible for tissue damage; CD4+ T lymphocytes, particularly those of the Th2 subtype, orchestrate eosinophilic inflammation, although Th1 and Th17 subsets are also implicated in granulomatous inflammation and vascular injury [6].

Positivity to ANCA is reported to occur in about 40% of patients, most commonly with antibodies directed against myeloperoxidase, the so-called p-ANCA [7]. The presence or absence of ANCA defines distinct clinical phenotypes: ANCA+ patients tend to exhibit vasculitis manifestations such as glomerulonephritis, peripheral neuropathy, and purpura, whereas ANCA− patients are more likely to develop eosinophil-driven organ damage, particularly cardiovascular involvement and eosinophilic gastroenteritis [8,9].

Positivity for antinuclear antibodies (ANAs) is rarely discussed in association to EGPA [10].

It is reported that the clinical evolution of EGPA follows a classically described, but variably expressed, triphasic course: a prodromal phase dominated by asthma, chronic rhinosinusitis with nasal polyposis (CRSwNP), reported in over 90% of cases, an eosinophilic phase characterized by peripheral eosinophilia with eosinophilic infiltration of tissues, and a vasculitis phase involving systemic manifestations due to small-vessel inflammation [11,12]. Some patients may progress rapidly while others remain in the eosinophilic stage without overt vascular involvement, complicating timely diagnosis; eosinophil-rich inflammation remains a consistent hallmark across all disease phenotypes [13,14].

Cutaneous manifestations, which are not included in the EGPA classification criteria of the American College of Rheumatology (ACR), are frequently observed and reported in 23% to 68% of patients [3]. The most common dermatologic finding is palpable purpura [15,16]; other skin lesions include subcutaneous nodules [17,18], non-specific maculopapular eruptions, urticaria, and, less commonly, angioedema, petechiae, sterile pustules, livedo reticularis, and vesicles [19]. Differentiating EGPA-specific skin involvement from other dermatologic conditions is essential in the diagnostic workup especially given the variability and non-specificity of certain cutaneous lesions [20] and of p-ANCA testing [21].

Until recently, the cornerstone of EGPA treatment was based on systemic oral corticosteroids (OCS), often combined with immunosuppressive agents such as cyclophosphamide and/or rituximab; while effective, these regimens are associated with substantial long-term toxicity or relevant secondary hypogammaglobulinemia [22]. Significant changes in treatment strategies are ongoing after the approval for EGPA treatment of biologic drugs that target interleukin-5 (IL-5) or its receptor, Mepolizumab and Benralizumab [23]. Mepolizumab, 300 mg monthly subcutaneously, is reported to improve disease control, reduce OCS burden, and enhance patients’ outcomes [24,25].

We report a monocentric, one-year observational study on 20 consecutive EGPA patients referring to the Allergy and Clinical Immunology Clinic in the Department of Clinical and Experimental Medicine of Messina University, Italy, focusing on cutaneous manifestations of EGPA correlated to patients’ characteristics, other disease manifestations, and laboratory findings.

## 2. Results

**Figure 1:**
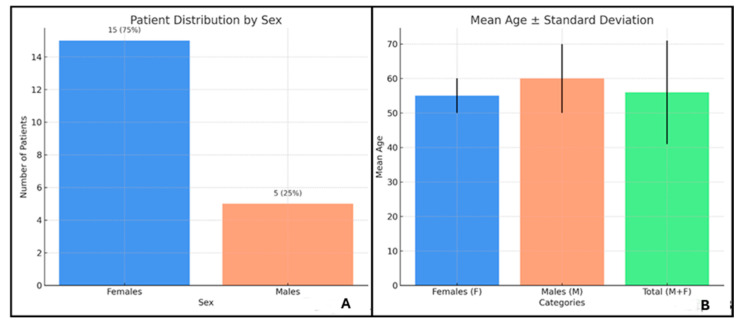
(**A**) shows patient’s distribution by sex; (**B**) reports mean age ± standard deviation.

The study population consisted of 20 EGPA patients (pts), 15 (75%) females (f) and 5 (25%) males (m), with a mean age of 56 ± 15 years. Among the 15 f, the mean age was 55 ± 16 years, while the 5 m had a mean age of 60 ± 9 years (Figure 1).

Blood eosinophils were elevated in all pts with a mean value of 2051 cells/mcL and were all under treatment with OCS with a mean daily dose of 25.66 mg. The BVAS was also elevated with a median score of 20.35.

**Figure 2:**
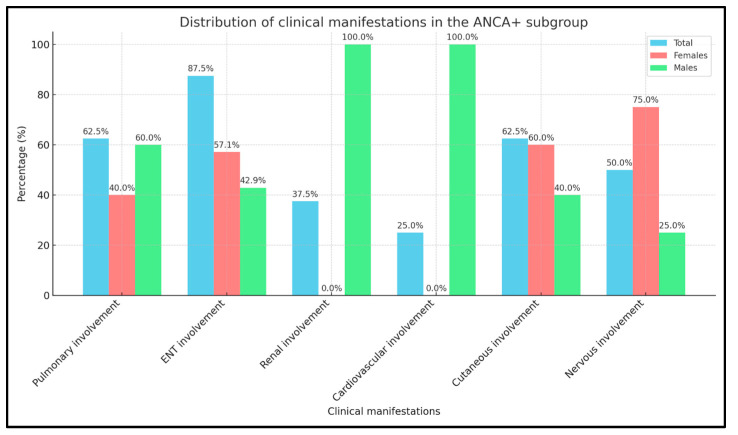
Distribution of clinical manifestations in p-ANCA+ females and males.

EGPA pts, according to the presence in blood tests of p-ANCA antibodies, or not, were divided into two groups. The ANCA+ group had 8/20 (40%) subjects, with an equal sex distribution of 4 f and 4 m, and a mean age of 60 ± 12 years. EGPA clinical features in this group included ear, nose, and throat (ENT) (4 f and 3 m, 87.5%), pulmonary (2 f and 3 m, 62.5%), renal (3 m, 37.5%), cardiovascular (2 m, 25%), and nervous system involvement (3 f and 1 m, 50%). Cutaneous manifestations were present in 3 f and 2 m (62.5%) (Figure 2).

**Figure 3:**
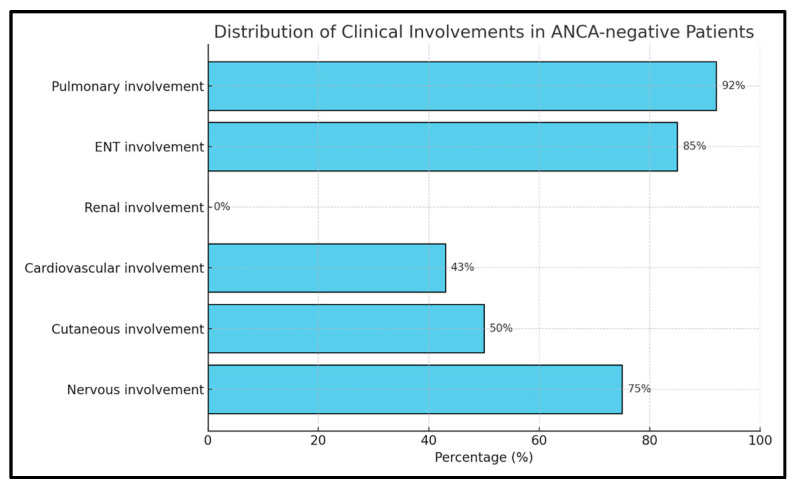
Distribution of clinical manifestations in the EGPA ANCA− group.

The ANCA− group of 12 subjects (60%) had a f prevalence, specifically 11 f and 1 m, with a mean age of 54 ± 16 years. EGPA clinical features in this group included ENT (9 f and 1 m, 83.3%), pulmonary (10 f and 1 m, 91.7%), cardiovascular (4 f and 1 m, 41.7%), and nervous system involvement (8 f and 1 m, 75%), but no renal involvement. Cutaneous manifestations were present in 5 f and 1 m (50%) (Figure 3).

### 2.1. Distribution of Clinical Features in ANCA+ and ANCA− EGPA Patients

We compared the distribution of clinical features (pulmonary, ENT, renal, cardiovascular, cutaneous, and nervous) between the two EGPA patients’ groups: ANCA+ and ANCA−. A Chi-square test was performed to assess whether there were statistically significant differences in the frequency of organ involvement between groups. Expected frequencies were calculated based on the marginal totals of the contingency table.

**Figure 4:**
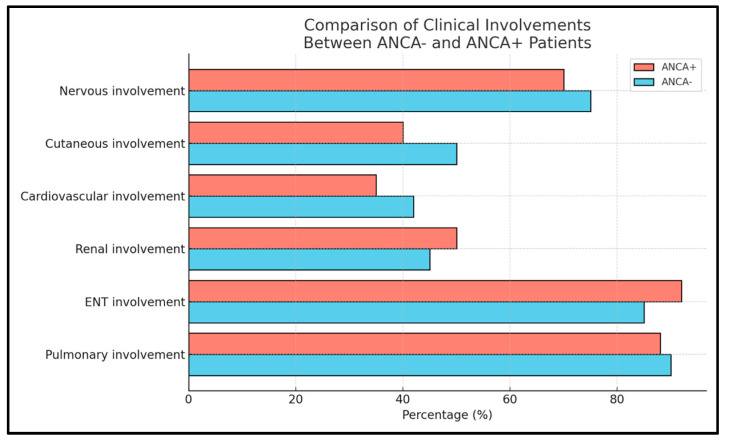
EGPA clinical involvement in ANCA- and ANCA+ patients.

The Chi-square value was 10.1 (df = 5, α = 0.05), which was lower than the critical value of 11.07, and therefore the null hypothesis could not be rejected. No statistically significant differences in clinical features between ANCA+ and ANCA− patients was found (Figure 4).

### 2.2. ANA Titration in ANCA+ and ANCA− EGPA Patients

ANA in EGPA pts’ blood samples were assessed using standard immunofluorescent immunological assays, with 10 pts testing positive (50%) and 10 negative (50%). When analyzing serological profiles combined with ANCA positivity or negativity, the following distribution was observed: ANA-positive/ANCA+: 2 pts (10%); ANA-positive/ANCA−: 8 pts (40%); ANA-negative/ANCA+: 6 pts (30%); and ANA-negative/ANCA−: 4 pts (20%) (Table 1).

### 2.3. Cutaneous Manifestations

**Figure 5:**
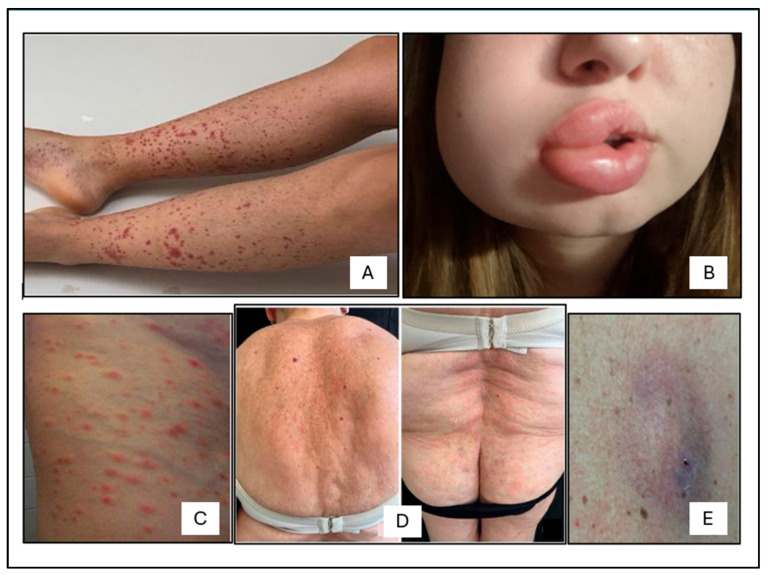
Photographic images of skin manifestations in EGPA patients: (**A**) purpura; (**B**) segmental angioedema; (**C**) urticarial vasculitis; (**D**) non-specific maculopapular rash; (**E**) subcutaneous nodule.

The 20 EGPA patients presented different types of cutaneous manifestations (Figure 5) that were observed in 5 ANCA+ (3 f, 2 m) and 6 ANCA− patients (5 f, 1 m). Cutaneous manifestations included purpura (Figure 5A) in 3 ANCA+ (1 f, 2 m) and 1 ANCA− patient (f); angioedema (Figure 5B) in 1 ANCA+ and in 2 ANCA− patients (all f); urticarial vasculitis (Figure 5C) in 1 ANCA+ and 1 ANCA− patient (both f); non-specific maculopapular rash (Figure 5D) in 1 ANCA− patient (f); and nodules (Figure 5E) in 1 ANCA− patient (m).

The skin manifestations had appeared in all patients at the eosinophilic stage of the disease. To assess whether there were significant differences in the distribution of specific skin manifestations between ANCA+ and ANCA− patients, due to the small sample size, Fisher’s exact test was used.

For each cutaneous manifestation 2 × 2 contingency tables were constructed to compare the presence or absence of the manifestation between the two groups.

**Figure 6:**
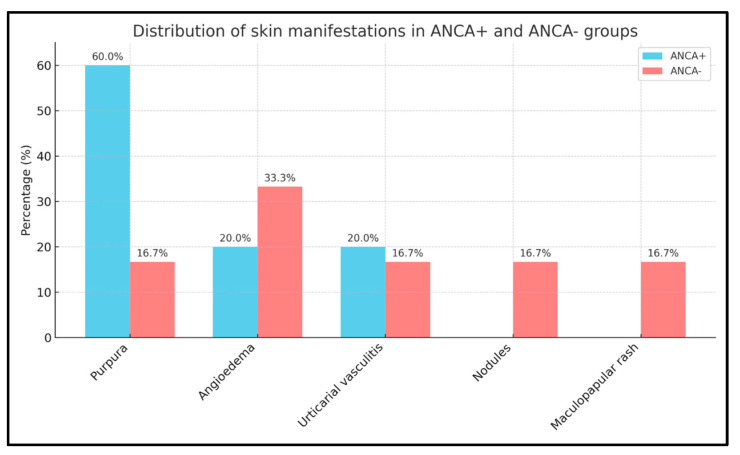
Cutaneous manifestations in ANCA+ and ANCA− patients.

The analysis revealed that none of the skin manifestations showed a statistically significant difference between ANCA+ and ANCA− patients. However, purpura appeared to be more frequent among ANCA+ patients, with a *p*-value of 0.242 (Figure 6).

In one ANCA+ f-patient, skin involvement with periorbital segmental angioedema was the only objective sign of EGPA relapse. This patient, after the diagnosis of EGPA, was started on Mepolizumab, 300 mg/monthly, as add on treatment to Prednisone 5 mg daily. Four months after starting Mepolizumab treatment the patient had no systemic symptoms and had become ANCA−; but 2 months after weaning prednisone treatment, she complained of fatigue, chronic headache, and periorbital edema, with laboratory evidence of increased inflammatory markers and reappearance of p-ANCA positivity with normal blood eosinophils. After another course of Prednisone, 5 mg for a month and continuing Mepolizumab 300 mg treatment, she became asymptomatic and regained ANCA negativity, which was persisting after six months’ follow-up.

In one patient from the ANCA− group, before the EGPA diagnosis, a dermatologic consultation was requested due to the appearance of widespread, intensely pruritic papular skin lesions, partly with central excoriation and focal clustering over the extensor surfaces. The dermatologist recommended skin biopsy for diagnostic clarification. Histopathological analysis of the punch biopsy revealed a predominantly neutrophilic infiltrate with abundant eosinophils, vascular wall necrosis, and leukocytoclastic findings consistent with urticarial vasculitis.

This finding, integrated with additional clinical evidence, such as poor asthma control, the presence of pulmonary nodules, and ground-glass opacities on chest CT, supported the diagnosis of EGPA. Initiation of treatment with Mepolizumab, 300 mg every 4 weeks, led to marked improvement in respiratory symptoms and complete resolution of cutaneous manifestations, with no relapse observed during the 12-month follow-up period.

### 2.4. Descriptive Analysis of Biomarkers, BVASs, and OCS Intake Correlated to Anti-IL5 Treatment

**Figure 7:**
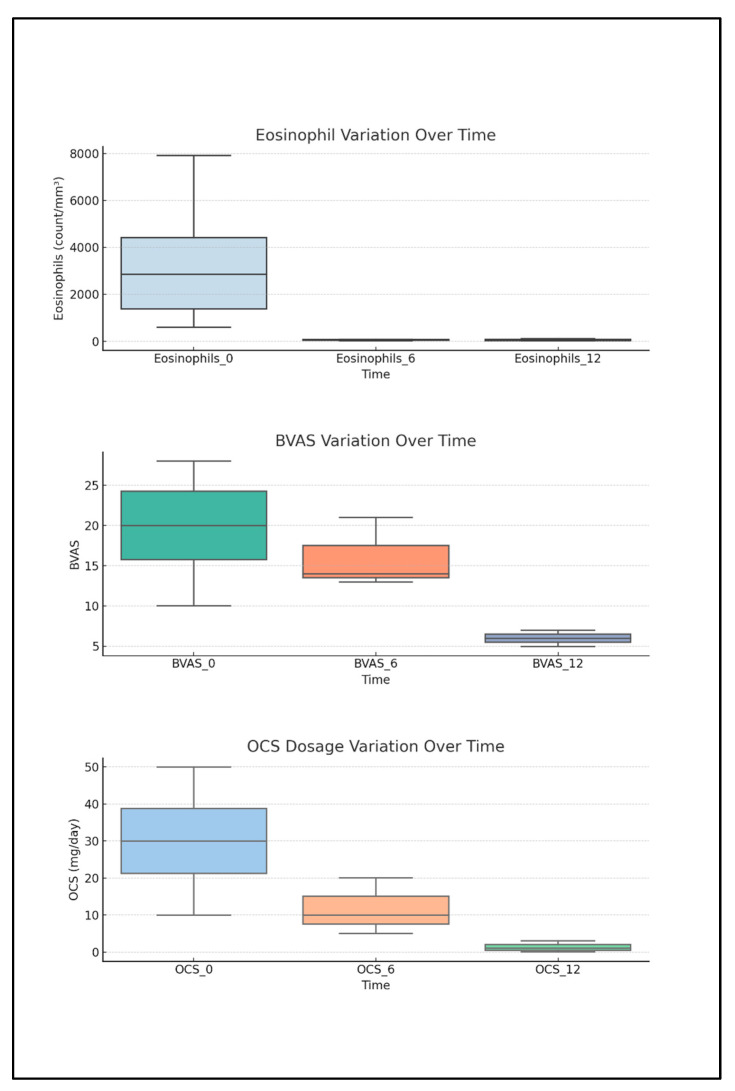
Progressive reduction in blood eosinophils, BVASs, and OCS dosage after starting Mepolizumab, a 300 mg monthly treatment at 6 and 12 months.

During the follow-up period on Mepolizumab, mean blood eosinophil counts decreased markedly from 2051/mm^3^ at baseline to 95/mm^3^ at 6 months and stabilized at 74/mm^3^ at 12 months. Similarly, the Birmingham vasculitis score (BVAS) dropped from a mean of 20.35 at baseline to 11.25 at both 6 and 12 months. OCS dosage also showed a substantial decline, from a mean of 25.66 mg/day at baseline to 8.75 mg/day at 6 months and 2.89 mg/day at 12 months. These trends indicate a progressive clinical improvement and a reduced need for OCS throughout the follow-up period (Figure 7). ANA serum levels, present only in 10 pts, remained positive even after Mepolizumab treatment as an aspecific marker of autoimmunity.

## 3. Discussion

The etiology of EGPA remains incompletely understood and is believed to involve a complex interplay between genetic predisposition and environmental triggers, causing immune dysregulation, heterogeneous clinical presentation, and variable immunopathological features [26,27,28,29]. Two key biochemical markers, ANCA and eosinophil blood levels, offer critical insights into the pathophysiology and clinical heterogeneity of the disease [30,31]. Eosinophilia, both in blood and tissues, is a hallmark of EGPA and reflects the underlying eosinophil-driven inflammation [32]. Peripheral eosinophil counts are commonly used as diagnostic thresholds and correlate with disease activity [33]. Eosinophils exert their pathological effects through the release of cytotoxic granule proteins, such as major basic protein (MBP), eosinophil peroxidase (EPO), and eosinophil cationic protein (ECP) contributing to tissue damage, endothelial injury, and the formation of granulomas [34]. In the skin, this translates into a wide array of lesions, from palpable purpura and nodules to more severe presentations such as sterile pustules and necrotic blisters [35]. The eosinophilic infiltrate observed in cutaneous biopsies is a critical diagnostic clue and often coexists with vasculitic features or granuloma formation [36], while ANCA antibodies, particularly those directed against myeloperoxidase (MPO p-ANCA), represent another important biochemical marker of EGPA, although not always present [37]. ANCA positivity can be correlated to cutaneous vasculitis causing small-vessel inflammation, neutrophil activation, and endothelial damage [9,38]; this is especially relevant in leukocytoclastic vasculitis seen in skin biopsies [39]. The interaction between ANCA-primed neutrophils and eosinophils plays a synergistic role in driving inflammation [24]. In particular, the phenomenon of “neutrophil priming”, where apoptotic or activated neutrophils express proteinase 3 (PR3) or MPO, triggers antigen presentation and Th1-mediated granuloma formation [40]. This contributes to chronic inflammation and may explain the presence of granulomas and necrosis in some severe cutaneous lesions [41]. In our one-year monocentric observational study, we analyzed the immunological and clinical profiles of 20 EGPA pts with a particular focus on cutaneous manifestations, and response to biologic therapy, specifically anti-IL-5 treatment with Mepolizumab. Special attention was devoted to the diagnostic and prognostic relevance of skin involvement, an aspect which sometimes is underestimated but that in our experience provided crucial clues to disease activity and therapeutic response. Cutaneous manifestations were documented in both groups; skin lesions included palpable purpura, urticarial vasculitis, angioedema, subcutaneous nodules, and non-specific maculopapular rashes. Skin manifestations are not frequently reported in EGPA; a literature review was conducted using the MeSH terms “Churg-Strauss” and “Skin Manifestations”. The search, limited to articles published in the last 10 years, and available in English, provided 15 articles. Following initial screening, four articles were excluded based on title relevance and other five after full-text review due to their lack of alignment with the objective of our research. Only six articles reported data on skin manifestations in EGPA patients, five referred to anecdotal case reports, and one on cutaneous manifestations in pediatric EGPA patients (Table 2).

In our patient sample skin manifestations were more prevalent in ANCA− patients, but purpura was predominantly observed in ANCA+ patients (1 f and 2 m), typical of ANCA-mediated small-vessel vasculitis. Angioedema was present in 3 f, 2 ANCA−, and 1 ANCA+, suggesting that mechanisms other than ANCA-mediated vasculitis, such as complement activation or eosinophilic infiltration, may contribute to skin involvement in these patients. Urticarial vasculitis was evenly distributed across both groups, while subcutaneous nodules and non-specific maculopapular rashes were seen only in ANCA− patients. Skin involvement was more common among women than men in both subgroups. This gender discrepancy may suggest a sex-related predisposition to cutaneous manifestations, though the high proportion of female patients in our cohort (75%) may represent a confounding factor. The presence of ANA positivity in 50% of patients in our small population does not fully elucidate its clinical relevance in EGPA, but in our opinion rather than a bystander, it may help clinicians to suspect EGPA, as a trait of the presence of an autoimmune disease. ANA positivity was associated with EGPA as reported to other autoimmune diseases [47], and stayed positive even after Mepolizumab treatment. It was present also in ANCA− pts and therefore in our population they were not correlated to a false positive titration [48]. Notably, a significant subset of pts exhibited discordant serologies, either ANA+ (10; 50%) but ANCA− (8; 66.7%) or ANA-negative (10; 50%) and ANCA+ (6; 75%), highlighting the importance of comprehensive autoantibody screening in the differential diagnosis of autoimmune conditions. Altogether, our data suggest that skin involvement in EGPA is common and clinically informative as in the patient where periorbital angioedema preceded systemic features, demonstrating the prognostic utility of skin findings in disease monitoring. Nonetheless, prolonged use of high-dose OCS or immunosuppressor agents for treating EGPA may suppress or obscure dermatological signs, highlighting the need for careful and proactive cutaneous evaluation. Our findings underscore the importance of understanding, diagnosing, and managing cutaneous manifestations for a comprehensive management of EGPA patients. The small sample size limits the generalizability of the findings, and the high proportion of female patients (75%) may introduce bias, as gender-specific differences in EGPA manifestations have not yet been thoroughly explored. Furthermore, the follow-up period of one year is relatively short for a chronic condition like EGPA. Longer-term data would strengthen the conclusions about treatment efficacy and relapse rates. 

## 4. Materials and Methods

An observational, prospective study was performed on 20 patients with EGPA diagnosed according to ACR criteria [49]. Participants were recruited between January and December 2024, consecutively, without applying exclusion criteria; comorbidities were not present. All participants provided written informed consent before inclusion in the study, after being acknowledged about the research objectives, privacy protection, and the use of anonymous data in compliance with the European General Data Protection Regulation (GDPR) 2016/679. The study was approved by the Ethics Committee of the University Hospital of Messina (Protocol number 16/19) and adhered to the ethical principles outlined in the Declaration of Helsinki (2013).

A one-year follow-up was conducted with data collection, at EGPA diagnosis, 6 and 12 months after initiating Mepolizumab, 300 mg monthly subcutaneous treatment.

Patients underwent a complete examination including evaluation of cutaneous manifestations. Analyses included a complete blood count to monitor serum eosinophil values, ANCA and ANA evaluation. To confirm the diagnosis of EGPA, the Birmingham vasculitis activity score (BVAS), a validated tool and the most widely accepted measure of disease activity in major studies of vasculitis [50], was submitted to all patients. The BVAS quantifies a patient’s disease activity correlating nine organ systems, one general, and eight tissue-specific, including the ear, nose, and throat (ENT), abdominal, nervous, mucous membranes/eyes, renal, cardiovascular, pulmonary, and cutaneous localization of the disease.

In addition, a literature review using the MeSH terms “Eosinophilic Granulomatosis with Polyangiitis” (EGPA) and “Skin Manifestations” was conducted to contextualize the clinical and histopathological findings and compare them with previously reported cases.

### Statistical Analysis

Statistical analysis assessed associations between clinical characteristics, treatment response, and cutaneous manifestations. Categorical variables (sex, skin involvement, organ involvement) were presented as absolute frequencies and percentages, while continuous variables (age, eosinophil levels) were summarized as means and standard deviations.

Differences between patient subgroups, i.e., clinical manifestation in p-ANCA positive (ANCA+) vs. p-ANCA negative (ANCA−) patients, with vs. without cutaneous manifestations, were evaluated by selecting the appropriate statistical test based on the data distribution and expected frequencies to ensure the validity of the results.

The Chi-square test was applied to assess differences in the distribution of major clinical manifestations (cardiovascular, renal, ENT, pulmonary, nervous) between ANCA+ and ANCA− patients under conditions where the assumptions for the Chi-square approximation were met.

Fisher’s exact test was used to evaluate whether the distribution of specific cutaneous manifestations differed significantly between p-ANCA+ and p-ANCA− subgroups. Given the small sample size and potentially low expected frequencies in some categories, Fisher’s test was selected as it provides exact *p*-values and is robust in the context of small or unbalanced samples. A *p*-value was calculated for each comparison using Fisher’s exact test, with a significance threshold set at *p* < 0.05.

## 5. Conclusions

Skin manifestations in EGPA patients can serve as diagnostic clues and early warning signs of relapses, particularly when integrated with immunological and laboratory data. The successful use of anti-IL-5 agents support the central role of eosinophilic inflammation in EGPA pathogenesis and provides a targeted, steroid-sparing therapeutic option.

The type and distribution of lesions may differ according to ANCA status and underlying pathophysiologic mechanisms, offering valuable insights into disease phenotyping and suggesting that ANCA status together with ANA and blood eosinophil levels may influence the clinical phenotype of EGPA patients.

## Figures and Tables

**Table 1:** ANA Positivity and negativity in EGPA pts and within ANCA− and ANCA+ groups.

Parameter	Total pts (*n* = 20)	ANCA− pts (*n* = 12)	ANCA+ pts (*n* = 8)
ANA positive (*n*, %)	10 (50%)	8 (66.7%)	2 (25%)
ANA negative (*n*, %)	10 (50%)	4 (33.3%)	6 (75%)

**Table 2:** Articles found in the literature search conducted using the MeSH terms “Churg-Strauss” and “Skin Manifestations,” limited to articles published in the last 10 years and available in English.

N.	Authors and Year of Publication	Type of Study	DOI	Title	Aims	Conclusions
1	Laura Calabrese et al., 2024 [42]	Case report	DOI: https://doi.org/10.23736/S2784-8671.24.07713-2.	Erythema annulare centrifugum as clinical manifestation of -eosinophilic granulomatosis with polyangiitis—PubMed	A rare case of superficial erythema annulare centrifugum (EAC), occurring in a patient with EGPA is reported highlighting the importance of recognizing figured erythemas as potential cutaneous manifestations of EGPA to improve diagnosis and management.	This case expands the known skin manifestations of EGPA by reporting superficial erythema annulare centrifugum. Recognizing figured erythemas in EGPA is important for accurate diagnosis and treatment. Further studies are needed to clarify their role and frequency.
2	Yamamoto Toshiyuki et al., 2024 [43]	Letter to the Editor	DOI: https://doi.org/10.1097/DAD.0000000000002763	A Complex Vasculitis: Thrombophlebitis, Subcutaneous Granulomatous Arteritis, and Eosinophilic Granulomatosis With Polyangiitis Presenting Clinically as Livedo Racemosa With Nodular Erythema—PubMed	It describes a rare case of EGPA characterized by the coexistence of dermo-subcutaneous junctional thrombophlebitis and deep subcutaneous granulomatous arteritis within the same biopsy specimen, and highlights the diagnostic value of excisional biopsy in EGPA patients presenting with livedo racemosa and nodular erythema.	This case underscores the critical role of deep excisional biopsy in EGPA patients presenting with livedo racemosa (reticularis), nodular erythema, or subcutaneous nodules, as it is essential for identifying the diagnostic hallmark of granulomatous arteritis.
3	Aleksandra Fratczak et al., 2022 [44]	Case report	DOI not available	Torasemide-induced Vascular Purpura in the Course of Eosinophilic Granulomatosis with Polyangiitis—PubMed	A case of EGPA with cutaneous vasculitis potentially triggered by torasemide is described, emphasizing the role of drug exposure in disease onset.	This is the first case report on torasemide-induced vascular purpura in the context of EGPA. The temporal association between torasemide initiation and symptoms’ onset suggests the drug may act as an aggravating factor in latent EGPA. Careful evaluation of recent drug exposure is crucial in all cases of new-onset vasculitis to improve diagnosis and outcomes.
4	Catherine Bridges et al., 2020 [45]	Review	DOI: https://doi.org/10.1111/pde.14144	Cutaneous manifestations of childhood (cEGPA): A case-based review—PubMed	Clinical and histopathological characteristics of skin involvement in pediatric EGPA is described.	The high prevalence of skin involvement in cEGPA highlights the importance of dermatologic expertise in the early detection for a prompt recognition, despite diagnostic complexity, to optimize therapeutic success in affected children.
5	Rie Shiiyama et al., 2019 [46]	Case report	DOI: https://doi.org/10.1097/DAD.0000000000001451	A Case of Cutaneous Arteritis Presenting as Infiltrated Erythema in Eosinophilic Granulomatosis With Polyangiitis: Features of the Unique Morphological Evolution of Arteritis as a Diagnostic Clue—PubMed	A rare case of EGPA with cutaneous lesions involving different stages of subcutaneous muscular vessel vasculitis is presented, highlighting the diagnostic significance of eosinophilic and granulomatous arteritis in the same artery.	This case shows that the coexistence of eosinophilic and granulomatous arteritis within a single subcutaneous muscular artery can serve as a unique and valuable histopathological clue for diagnosing EGPA, especially in the context of cutaneous involvement.
6	Camila Carneiro Marques et al., 2017 [35]	Case report	DOI: https://doi.org/10.1590/abd1806-4841.20175522	Cutaneous manifestations of Churg-Strauss syndrome: key to diagnosis—PubMed	A case of a female patient affected by EGPA with important systemic manifestations and not very florid skin lesions is described.	Recognition of skin lesions by the dermatologist was essential for the clinical suspicion and confirmation of diagnosis, which allowed adequate treatment, reducing morbidity and contributing to prevent irreversible lesions in vital organs.

## Data Availability

Data supporting reported results are available if requested.

## Abbreviations

The following abbreviations are used in this manuscript:

EGPA	Eosinophilic granulomatosis with eosinophilia
PTS	Patients
F	Females
M	Males
ANCA	Anti-neutrophil cytoplasmic antibody
ANA	Antinuclear antibodies
BVAS	Birmingham vasculitis activity score
